# Herpes zoster prevalence following epidural steroid injections: a retrospective review

**DOI:** 10.1016/j.inpm.2025.100597

**Published:** 2025-05-29

**Authors:** Laura Furtado-Pessoa-de-Mendonca, Sebastian Encalada, Alejandro Hallo-Carrasco, Johanna Mosquera-Moscoso, Matthew A. Cascio, Robert Pagan-Rosado, Michael D. Osborne, Jason S. Eldrige, Christine L. Hunt

**Affiliations:** a Department of Anesthesiology and Perioperative Medicine, Mayo Clinic, Jacksonville, FL, USA; b Department of Pain Medicine, Mayo Clinic, Jacksonville, FL, USA; c Department of Family Medicine, Mayo Clinic, Jacksonville, FL, USA

**Keywords:** Herpes zoster, Epidural steroid injection, Post-steroid injection complications, Infection, Chronic pain

## Abstract

**Introduction:**

Herpes Zoster, or shingles, is an infection caused by the reactivation of the latent Varicella zoster virus within a sensory ganglion, leading to painful skin lesions localized along dermatomes. Patients undergoing pain medicine procedures involving steroids may face an elevated risk of shingles, which can significantly impact their quality of life. Though rare, HZ has been reported following minimally invasive procedures, such as epidural steroid injections.

**Objectives:**

We evaluated the prevalence of shingles within 31 days after epidural steroid injections within Mayo Enterprise sites.

**Methods:**

A retrospective chart review included all patients who reported a new HZ event within 31 days after receiving an epidural steroid injection. Information on patient demographics, procedure details, and potential risk factors for herpes zoster was assessed using qualitative analysis.

**Results:**

A total of 50,270 epidural injections were performed during the analyzed period. After initial screening, 149 patients were included for chart review, and 37 individuals met the inclusion criteria. Within this subgroup, the median age was 72, and 21 patients were female (56.76 %). The mean timeframe from the procedure until onset of symptoms of infection as reported in the medical record was 15.9 days. Among the patients in the study, 24 patients (64.86 %) had an identified immunocompromised status, and 28 (75.68 %) had an incomplete vaccination status at the time of infection.

**Conclusion:**

The incidence of HZ following ESI is low. Other risk factors linked to HZ were identified in our cohort, confounding a possible causal relationship. Prospective studies are needed to elucidate any relationship between ESI and HZ.

## 1 Introduction

Herpes Zoster (HZ), commonly known as shingles, is a viral infection triggered by the reactivation of the dormant Varicella zoster virus (VZV) within a nerve root, manifesting as painful skin lesions localized along specific dermatomes or areas of the skin [1,2]. Shingles most often occurs in patients over 50 years old and in those with conditions linked to an immunocompromised status (e.g., autoimmune disorders, transplant recipients, acquired immunosuppression, malignancy) [3]. Corticosteroids with systemic administration have also been identified as potential contributors for HZ, especially within the first month after steroid exposure [4]. While the risk of HZ with localized agents for intraarticular anti-inflammatory injections appears low, the risk associated with central steroid injections, such as epidural steroid injections (ESIs), is less clear [[5], [6], [7]]. Epidural steroid injections are a common therapeutic modality employed in pain management to mitigate symptoms and reduce inflammation associated with chronic radiculopathy. These injections involve the administration of corticosteroids directly into the epidural space [8]. Although a rare occurrence, documented cases of HZ following ESIs suggest a potential link between the theoretical direct suppression of local immune defenses and viral reactivation, as the virus may reside dormant in the dorsal root ganglion [5]. However, most information regarding viral infection after ESI administration derives from limited data pools, including case reports and case series [9,10].

The objective of this study is to assess the incidence of HZ within 31 days following an ESI. Understanding this relationship is paramount to developing effective strategies for risk mitigation and improving patient care.

## 2 Methods

The study was deemed exempt from Institutional Review Board review (IRB#23–001438). We performed a retrospective chart review including the records of individuals with a described episode of HZ within 31 days after receiving an ESI performed from January 1st, 2019, to September 30th, 2024, subject to best data availability. The data was screened using a self-service reporting tool of the electronic medical record (Epic Systems Corporation, HYPERSPACE, November 2022). The search strategy was designed to include data from all Mayo Clinic sites. We included “spinal injection” and “spinal steroid injection” as keywords to appraise how many ESIs have been performed within the time frame. These terms also incorporated caudal steroid injections. Search parameters were further limited to "herpes zoster infection" and "31-day" interval.

Research staff manually reviewed the pertinent records identified by the reporting tool, and relevant data was extracted, including demographic information, comorbidities, medication, procedure details, HZ vaccination status, evidence of immunosuppressive status, description(s) of the HZ lesions, and the procedure/technique used for the injection. We considered the HZ rash distant from the injection site if it occurred more than five dermatome levels away. Additionally, we set the period between the procedure and the onset of symptoms from 0 to 31 days. Although, shingles can occur beyond that period, [4,9], we set our time frame to limit the influence of secondary treatments on the onset of infection. We classified vaccination status as incomplete if the patient had less than two doses of the Shingrix vaccine. Patients with commorbities such as as malignancy, transplant, autoimmune disorder, or chronic kidney disease were considered as immunocompromised. We reviewed the medication list to identify drugs with potential immunomodulatory effects that could contribute to HZ infection.

We performed descriptive analysis using Excel to analyze the data qualitatively. The study adhered to ethical guidelines, maintaining patient confidentiality and privacy throughout the data collection and analysis processes.

## 3 Results

**Fig. 1:**
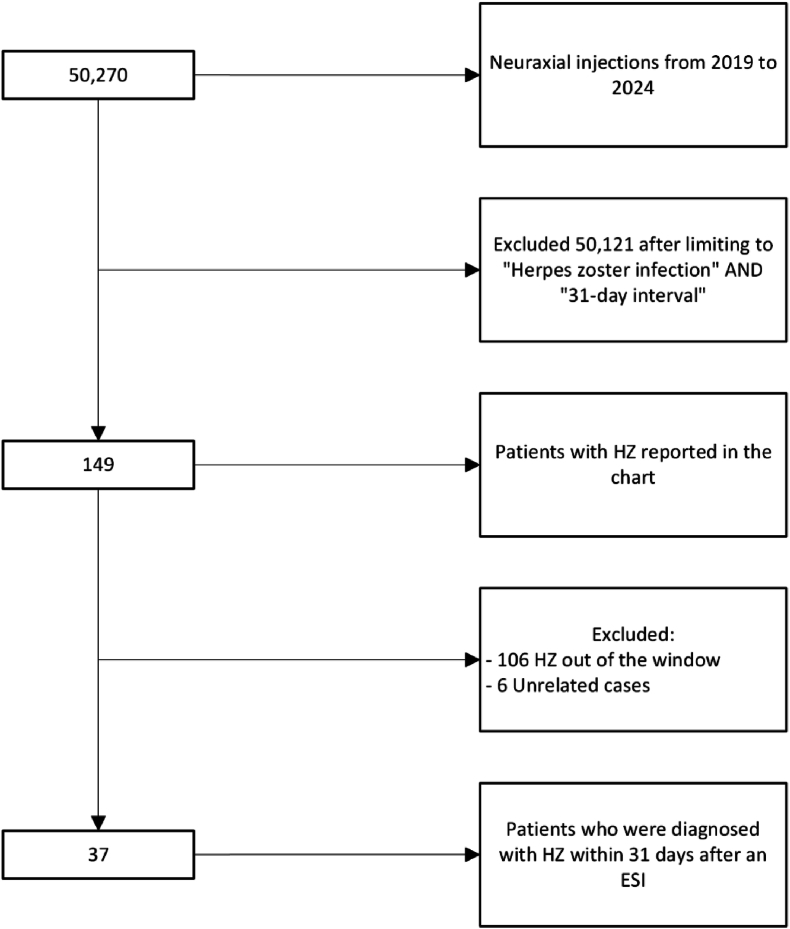
Record selection flow chart.

We identified 50,270 epidural injections performed throughout the Mayo Clinic Enterprise during the specified time points and included 149 patients with HZ reported on the chart after limiting the search to previously mentioned keywords. Among these, 112 individuals were excluded for developing shingles out of the 31-day window or for not matching other inclusion criteria. Thirty-seven patients were included for analysis (see Fig. 1). Within this subgroup, 21 patients were female (56.76 %), 16 were male (43.24 %), and the age range varied from 34 to 90+, with a median age of 72 years-old. More detailed demographic information is described in Table 1.

**Table 1:** Demographic information.

	N (%)
Gender	
Female	21 (56.75)
Male	16 (43.24)

**Race/Ethnicity**	
White-Caucasian	35 (94.60)
Black or African American	1 (2.70)
American Indian	1 (2.70)

**Age group**	
30–49	4 (10.8)
50–69	13 (35.13)
70–90+	20 (54.05)

The epidural approach was categorized as transforaminal, interlaminar or caudal, and the stratified incidence rate is described in Table 2. The lumbar region was most frequently targeted (n = 24; 64.86 %), followed by cervical (n = 5; 13.51 %), sacral (n = 5; 13.51 %), and thoracic (n = 3; 8.11 %). Radicular pain was the most frequent indication for ESI, observed in 31 patients (83.78 %), followed by "low back pain" (n = 2; 5.41 %), "spondylosis" (n = 2; 5.41 %), "spinal stenosis" (n = 1; 2.70 %), and "sciatica" (n = 1; 2.70 %). The most frequent steroid preparation was dexamethasone 10 mg (n = 19; 51.35 %), followed by betamethasone 6 mg (n = 7; 18.92 %) and methylprednisolone 80 mg (n = 7; 18.92 %). Other formulations included bethamethasone 12 mg (n = 2, 5.41 %), dexamethasone 5 mg (n = 1, 2.70 %) and dexamethasone 16 mg (n = 1, 2.70 %).

**Table 2:** HZ incidence rate per epidural approach.

Approach	Rate % (N)
Caudal	8.11 (3)
Interlaminar	40.54 (15)
Transforaminal	51.35 (19)

The timeframe until infection ranged from 2 to 31 days, with a mean of 15.9 days. The most common sites for infection sites were the thoracic and lumbar regions, accounting for 21.62 % (n = 8) each, followed by gluteal area and face 13.51 % (n = 5) each, and the sacral region 10.81 % (n = 4). Other affected areas included the cervical region, vulva, and shoulders (n = 2, 5.41 %) each, and upper extremity (n = 1, 2.70 %). Nine patients (24.32 %) developed shingles at a site considered distant from the procedure injection. Table 3 details previous information according to each patient.

**Table 3:** Description of procedure and infection site.

Patient #	Steroid Preparation/Dosage (mg)	Technique/Level of Injection	Infection site
1	Methylprednisolone/80	IL/T11-12	Thoracic
2	Dexamethasone/16	TF/L4-5	Thoracic
3	Methylprednisolone/80	TF/L5-S1	Shoulder∗
4	Dexamethasone/10	TF/C5-6	Face
5	Dexamethasone/10	TF/S1	Gluteal
6	Betamethasone/6	IL/L4-5	Gluteal
7	Dexamethasone/10	IL/C6-7	Vulva∗
8	Betamethasone/6	TF/L5	Face∗
9	Dexamethasone/10	TF/L5	Sacral
10	Betamethasone/6	IL/L1-2	Gluteal
11	Betamethasone/6	IL/L4-5	Sacral
12	Dexamethasone/10	TF/L3-4	Sacral
13	Dexamethasone/10	TF/L5	Lumbar
14	Betamethasone/6	IL/L4-5	Gluteal
15	Dexamethasone/10	TF/S1	Lumbar
16	Dexamethasone/10	IL/L5-S1	Cervical∗
17	Dexamethasone/10	TF/L4	Thoracic
18	Dexamethasone/10	TF/C7	Arm
19	Betamethasone/6	IL/L3-4	Thoracic
20	Dexamethasone/10	TF/C5-6	Thoracic
21	Methylprednisolone/80	IL/L5-S1	Gluteal
22	Betamethasone/6	IL/L4-5	Cervical∗
23	Dexamethasone/10	IL/C6-7	Thoracic
24	Dexamethasone/10	TF/L5	Thoracic
25	Dexamethasone/10	TF/L2-3	Face∗
26	Methylprednisolone/80	IL/L4-5	Thoracic
27	Methylprednisolone/80	IL/L4-5	Vulva
28	Methylprednisolone/80	IL/L5-S1	Sacral
29	Betamethasone/12	Caudal/Sacral hiatus	Lumbar
30	Dexamethasone/10	TF/L5-S1	Lumbar
31	Methylprednisolone/80	IL/T3-4	Face∗
32	Dexamethasone/10	TF/T4	Lumbar
33	Dexamethasone/10	TF/L3-4	Face∗
34	Dexamethasone/10	Caudal/Sacral hiatus	Lumbar
35	Dexamethasone/10	TF/L5	Shoulder∗
36	Betamethasone/12	Caudal/Sacral hiatus	Lumbar
37	Dexamethasone/5	TF/L2	Lumbar

IL, interlaminar; TF, transforaminal; ∗ Manifestations considered distant from the injection site.

**Fig. 2:**
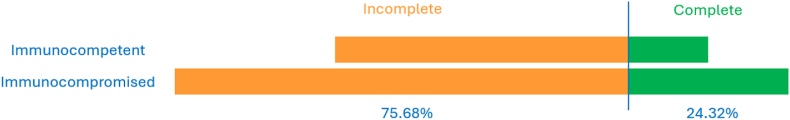
Correlation between vaccination status and immune system health.

Among the cohort, twenty-four patients (64.86 %) had an identified immunocompromised status, and 28 individuals (75.68 %) had an incomplete vaccination status at the time of the infection (see Fig. 2). Twenty-eight patients (75.68 %) – not necessarily the same as those with incomplete vaccination – were taking medications known to have an immunosuppressive effect (e.g. calcineurin inhibitors, antineoplasic agents, antirheumatic drugs, JAK inhibitors, statins, steroids).

## 4 Discussion

This retrospective study reports the identified incidence of HZ within thirty-one days after ESIs for pain indications. We found an incidence of 0.074 % (37 cases out of 50,270 injections), which is lower than what has been reported in other studies that have estimated the overall risk of infection (including viral, bacterial, and fungal) after spinal procedures [9,10]. The rate of infection of this retrospective study may well be under-reported, as patients may have developed HZ but not reported the event to their care team or it was otherwise not documented in the medical record. The lack of similar studies limits our ability to compare our estimated incidence, making our study unique.

Epidemiological trends have shown an increased prevalence of HZ in elderly Caucasian females, a trend also reflected, though on a small scale, in our findings [3,11].

Eighty-nine percent of our cohort was over 50 years old, 64.86 % of the patients had an identifiable immunocompromised status, and 75.68 % were taking medications known for their immunosuppressive effects. While systemic corticosteroid administration have been associated with increased risk of zoster [4], the specific risk from ESIs remains unclear. In our cohort, the overall incidence appears to be low, and direct causation of course cannot be proven with a retrospective analysis.

Many factors, including the site of delivery, type of corticosteroid, and dose regimen, could contribute to the immunomodulatory effects of ESIs, leading to a possible reactivation of dormant VZV [12]. We identified two main injection approaches in our cohort: transforaminal epidural steroid injection (TFESI) and interlaminar epidural steroid injection (ILESI). The TFESI approach deposits medication directly around the dorsal root ganglion, where HZ may lie dormant, potentially facilitating a more local/direct immunomodulatory effect from the delivered steroid relative to an ILESI [13,14]. Conversely, given the epidural vascularity, ILESIs can have a higher systemic absorption of steroids when compared to TFESI technique [15].

Most patients in our cohort developed a pathologic rash near the injection site. Specifically, twenty-five patients (75.67 %) experienced HZ in a proximal dermatome, while nine patients (24.32 %) developed shingles in dermatomes distant from the injection site (e.g. a patient who received a C6-C7 injection later presented with HZ in the vulvar region). This distribution was observed with similar proportions across both injection approaches. In the TFESI group, fourteen (73.68 %) had proximal HZ and five (26.32 %) had distant HZ. Similarly, in the ILESI group, eleven (73.33 %) of cases were proximal HZ and four (26.67 %) experienced distant shingles. Studies have reported the systemic effect of local steroid injections due to the extension of the medication beyond the intended area [16]. However, the predominance of proximal cases in our study supports the idea that HZ may be more attributable to the direct spread of steroid medication via injection rather than a systemic immunomodulatory effects of steroids.

A significant proportion of our cohort had identifiable risk factors for shingles, including immunocompromised status, incomplete HZ vaccination and medications with known immunossupressive effects. These findings raise the question of whether completion of HZ vaccination should be considered as part of pre-procedural assessment, particularly in high risk patients undergoing elective ESI. Although the recombinant HZ vaccine is approved for both immunocompetent and certain immunocompromised patients, the ideal timing and efficacy pre-procedure is unknown. In elective settings, providers may consider engaging patients in shared decision-making discussions considering risks and benefits of vaccination prior to ESI.

Overall, HZ can be attributed to a number of risk factors, several of which were observed in our cohort, including immunocompromised status (concomitant use of immunosuppressive medications, incomplete vaccination status), and older age [11]. While these factors likely contributed to HZ incidence, the possibility that ESIs may confer an additional risk for shingles or be a direct contributor, as evidenced by the proximity of most dermatomal rashes to the injection site within a short timeframe, warrants further investigation.

### 4.1 Limitations

The study is a single-center analysis, which limits the generalizability of the findings to the larger population of patients who receive ESIs. This study's retrospective and descriptive nature and the small cohort analyzed limit our ability to posit the potential links between ESIs and HZ. However, it suggests that if there is a risk of HZ after ESI, that risk is likely small, and may be higher in older patients with immunocompromised status. A significant limitation of the available data was that most patients meeting inclusion criteria were Caucasian (94.60 % of the cohort identified as Caucasian). Although selection bias seems to be a concern, our selection of participants was based on a digital automated software of electronic medical records limited to only clinical criteria.

## 5 Conclusion

The overall incidence of HZ after 31 days following ESIs was very low in this retrospective analysis. A significant proportion of our cohort also presented other well-established risk factors for HZ. This coexistence of multiple potential risk factors obscures our ability to theorize possible direct associations. Prospective studies are warranted to more accurately describe the risk of HZ reactivation following ESI.

## Funding

This research did not receive any specific grant from funding agencies in the public, commercial, or not-for-profit sectors.

## Declaration of competing interest

The authors declare the following financial interests/personal relationships which may be considered as potential competing interests: Christine L. Hunt reports a relationship with 10.13039/100019969Nevro Corp that includes: funding grants. If there are other authors, they declare that they have no known competing financial interests or personal relationships that could have appeared to influence the work reported in this paper.
